# A Comprehensive, Multisystemic Early Childhood Program and Obesity at Age 37 Years

**DOI:** 10.1001/jamapediatrics.2020.6721

**Published:** 2021-03-22

**Authors:** Arthur J. Reynolds, Lauren Eales, Suh-Ruu Ou, Christina F. Mondi, Alison Giovanelli

**Affiliations:** 1 Human Capital Research Collaborative, Institute of Child Development, University of Minnesota, Minneapolis; 2 Brazelton Touchpoints Center, Boston Children’s Hospital, Harvard University, Boston, Massachusetts; 3 Department of Pediatrics-Adolescent Medicine, University of California, San Francisco

## Abstract

This cohort study analyzes reductions in obesity rates at age 37 years among individuals who participated in a multisystemic Child-Parent Center preschool program.

Given the scarcity of effective obesity prevention programs, there is a pressing need for innovative and scalable models that begin early in life and break risk cycles. Promising evidence exists that multisystemic preschool programs can promote healthy body mass and positive behaviors,^1,2^ but to our knowledge, no existing or large-scale programs have assessed long-term links between such programs and health outcomes in midlife. We assessed the association of participation in an evidence-based, multicomponent Child-Parent Center (CPC) preschool program in Chicago, Illinois,^3^ with body mass and obesity at age 37 years for a sample of predominately Black individuals who grew up in poverty.

## Methods

The Chicago Longitudinal Study (CLS) used a prospective matched-group quasi-experimental design of 1539 participants born in 1979 and 1980, including 989 children who participated in a CPC at ages 3 or 4 years,^4^ and a comparison group of 550 children who attended randomly selected schools with the usual intervention of full-day kindergarten without preschool. All participants resided in high-poverty areas wherein more than 90% of the residents were Black (eMethods in the Supplement). From ages 32 to 37 years (August 20, 2012, to July 18, 2017), 1104 participants (71.7%) completed a survey on health and well-being. To corroborate and extend the survey data, an in-person health examination was completed for a subsample of 301 participants (19.6%) from ages 37 to 39 years (March 24, 2017, to December 21, 2019) at University Feinberg School of Medicine in Chicago, Illinois. Data were analyzed from July to September 2020. The study was approved by the institutional review board of the University of Minnesota. Health. Examination data were approved by the institutional review boards of the Northwestern University Feinberg School of Medicine and the University of Minnesota. Informed consent was written and oral.

Based on responses to height and weight questions, we assigned body mass index (BMI; calculated as weight in kilograms divided by height in meters squared) scores to 1042 participants (689 from the CPC participation group and 353 from the comparison group) and compared these scores with the examination data using Pearson correlation. We created 3 variables for obesity prevalence: any obesity (BMI of 30.0 or higher), moderate obesity or higher (BMI of 35.0 or higher), and severe obesity (BMI of 40.0 or higher). Scores correlated highly with examination data (*r* = 0.85).

The goal of the CPC program is to promote health and education equity through comprehensive educational enrichment, family support, health resources, and community outreach services. Key elements include small classes, individualized learning experiences, parenting classes on health and nutrition, support groups, and community engagement (eMethods in the Supplement).^5^ After 1 or 2 years of part-day preschool, kindergarten through third grade services are provided.

The model was estimated by linear and probit regression with inverse propensity score weighting to adjust for differential attrition. The covariates were CPC school-age participation from first to third grade and 17 multilevel baseline characteristics, including birthweight, gender, sex, neighborhood characteristics, and family sociodemographic risk factors (eMethods in the Supplement).^4^ Analyses were conducted using SPSS version 26 (IBM). *P* values were 2-tailed, and significance was set at *P* < .05. Moderators included sex; high neighborhood poverty, defined as 40% or more of the population at or below the federal poverty line; and family risk, defined as meeting 4 or more of 8 risk factors.^1,2,4^

## Results

Of 1104 respondents, 601 (54.4%) were female. The mean (SD; range) age at completion was 34.9 (1.4; 31.4-37.8) years. The mean (SD) BMI was 30.4 (6.8). For the population of 1042 participants with BMI data, there were 468 participants (44.9%) with any obesity, 208 (20.0%) with moderate obesity or higher, and 89 (8.5%) with severe obesity. Moderate obesity alone (119 individuals [11.5%]) was not analyzed. High neighborhood poverty and family risk were associated with higher BMI levels. As shown in the Table, CPC participation was associated with significantly lower BMI (adjusted difference, −1.0%; *P* = .04; standardized difference, −0.15). The pattern for moderate obesity or higher was similarly inverted. All CPC high-risk subgroup members showed significant reductions in BMI and obesity. The largest reductions were among female individuals (adjusted difference, −16.6%; *P* < .001) and those in high-poverty neighborhoods (adjusted difference, −15.3%; *P* = .001). No differences by CPC participation were found for male individuals, lower neighborhood poverty rates, and lower family risk. Significant moderator effects between groups were detected for female individuals and participants from high poverty neighborhoods. Standardized mean difference for BMI used the mean square residual (6.65) of the unweighted regression; mean differences for other covariates were from probit transformation of proportions.

**Table.** Means, Rates, and Inverse Propensity Score Weighting–Adjusted Differences in Body Mass Index (BMI)^a^ Outcomes at Age 37 Years for Child-Parent Center (CPC) Participants and Comparison Group Participants in the Chicago Longitudinal Study^b^

Outcome	%				Adjusted difference, percentage points	*P* value	Standardized difference	Reduction in BMI category relative to comparison group, %
	CPC (n = 689)		Comparison (n = 353)					
	Unadjusted	Adjusted	Unadjusted	Adjusted				
**Total sample (n = 1042)**								
BMI, mean	30.2	30.0	30.8	31.0	−1.0	.04	−0.15	3.2
Obesity								
Any (≥30.0)	44.0	43.4	46.7	47.9	−4.5	.13	−0.13	9.4
Moderate (≥35.0)	19.3	18.8	21.3	22.9	−4.1	.09	−0.14	17.9
Severe (≥40.0)	8.6	8.0	8.5	9.7	−1.7	.32	−0.13	17.5
**Female (n = 543)**								
BMI, mean	31.0	30.8	32.7	33.3	−2.5	.002	−0.38	7.5
Obesity								
Any (≥30.0)	46.8	45.9	59.2	62.5	−16.6	<.001	−0.41	26.6
Moderate (≥35.0)	25.4	24.8	31.4	31.0	−6.2	.08	−0.17	20.0
Severe (≥40.0)	13.1	12.2	13.0	13.7	−1.5	.60	−0.09	10.9
**High-poverty neighborhood (n = 518)**								
BMI, mean	30.2	30.1	32.0	32.1	−2.0	.008	−0.30	6.2
Obesity								
Any (≥30.0)	43.9	41.6	55.7	56.9	−15.3	.001	−0.38	26.9
Moderate (≥35.0)	20.2	19.9	21.3	27.8	−7.8	.03	−0.26	28.1
Severe (≥40.0)	9.6	9.7	12.2	12.4	−2.7	.32	−0.15	21.8
**High family risk status (n = 742)**								
BMI, mean	29.9	29.9	31.3	31.3	−1.4	.01	−0.21	4.5
Obesity								
Any (≥30.0)	42.3	42.3	49.4	51.2	−8.9	.04	−0.23	17.4
Moderate (≥35.0)	18.8	18.2	23.8	25.5	−7.3	.04	−0.28	28.6
Severe (≥40.0)	9.4	8.7	8.7	10.3	−1.6	.53	−0.11	15.5

^a^ Body mass index was calculated as weight in kilograms divided by height in meters squared.

^b^ Values for male individuals, lower neighborhood poverty, and lower family risk were excluded. No difference by CPC participation were found for these subgroups. Significant moderator effects between groups were detected for female individuals and participants from high-poverty neighborhoods (defined as 40% or more of residents at or below the federal poverty line). Adjusted models included CPC school-age participation and 17 covariates and inverse propensity score weighting attrition. Among covariates were baseline characteristics including birthweight, gender, sex, neighborhood settings, and family sociodemographic risk factors (eMethods in the Supplement). Standardized mean difference for BMI used the mean square residual (6.65) of the unweighted regression; others were from probit transformation of proportions. For the population of 1042 participants with BMI data, there were 468 participants (44.9%) with any obesity, 208 (20.0%) with moderate obesity or higher, and 89 (8.5%) with severe obesity. Moderate obesity alone (119 individuals [11.5%]) was not analyzed.

The Figure displays means and obesity rates for subgroups. A compensatory pattern of benefits was found, wherein those with higher-risk characteristics or environments showed the largest reductions. Findings were robust with alternative models, including without inverse propensity score weighting adjustment.

**Figure.**
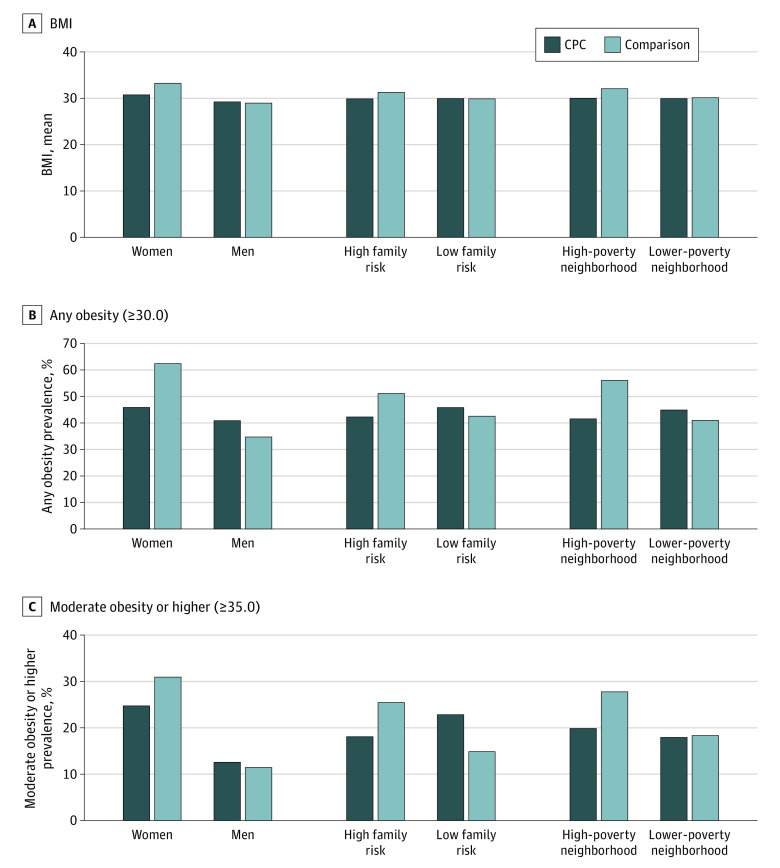
Body Mass Index (BMI) Outcomes at Age 37 Years for Subgroups of Child-Parent Center (CPC) Participants and Comparison Group Participants Values were adjusted for 17 baseline covariates, CPC school-age participation, and inverse propensity score weighting for attrition. High family risk was defined as 4 or more of 8 demographic risks. High-poverty neighborhood was defined as 40% or more of residents at or below the federal poverty line at baseline (1980 Census). BMI was calculated as weight in kilograms divided by height in meters squared.

## Discussion

As the first study, to our knowledge, of the association between participation in an existing large-scale preschool program and adult BMI, we found CPC participation was associated with significantly lower rates of adult BMI. This was especially apparent for high-risk groups, where reductions in obesity prevalence were up to 29%.

Increased priority on preventing obesity through early childhood programs^3^ can address health disparities exacerbated by existing socioeconomic inequities, such as multilevel poverty and segregation.^6^ The 20% to 30% reduction in obesity for those growing up in high-poverty neighborhoods suggests that comprehensive programs that engage families in multiple systems of education and care, such as CPC programs, can promote health across domains of well-being.^4,6^ The scope of this study, along with longer duration and an existing school-based structure, distinguish it from prior obesity prevention programs.^3^ A possible advantage is the focus on educational attainment,^5^ the leading social determinant of health in the US Department of Health and Human Service’s Healthy People initiative.

This study had limitations. As the study analyzed a comprehensive and high-quality program, results may not generalize to individuals who attended less advanced and comprehensive programs. BMI was self-reported, although examination scores correlated highly with self-reported data. In conclusion, a comprehensive school-based early childhood program showed evidence of improving healthy body mass for an urban and predominately Black cohort at a time of growing national need.

## Notes

Supplement. eMethods. eReferences.
