# Supplementary material for: A Comprehensive, Multisystemic Early Childhood Program and Obesity at Age 37 Years
Source: JAMA Pediatr. 2021 Mar 22;175(6):637–40. doi: 10.1001/jamapediatrics.2020.6721 (PMC7985813; doi:10.1001/jamapediatrics.2020.6721)
Supplement: Supplement. — eMethods. eReferences. [file jamapediatr-e206721-s001.pdf]

# Supplemental Online Content

Reynolds AJ, Eales L, Ou S-R, Mondi CF, Giovanelli A. A comprehensive, multisystemic early childhood program and obesity at age 37 years. *JAMA Pediatr*. Published online March 22, 2021. doi:10.1001/jamapediatrics.2020.6721

**eMethods.**
**eReferences.**

This supplemental material has been provided by the authors to give readers additional information about their work.

© 2021 Reynolds AJ et al. *JAMA Pediatrics*.

eMethods

**Body Mass Index Measurement**

Participants' Body Mass Index (BMI) was assessed by self-report of height and weight on the adult survey/interview from ages 32 to 37 (August 20, 2012 to July 18, 2017; mean age of 35 years). With an average length of 150 minutes and covering a wide range of topics, the survey/interview was completed via phone, online, in person, or mail-in. Participants were asked both for their height and weight at their current age while taking the survey, as well as what they believe their height and weight was at age 18. BMI is one general indicator of body fat and is calculated as height (in meters) divided by weight (in kilograms) squared. Overweight is defined as a BMI between 25 and 29.9 m/kg2 and obesity applies to those with a BMI of 30.0 m/kg2 or higher. Moderate and severe obesity is defined at the thresholds of 35.0 and 40.0, respectively. After performing this calculation with participant responses, 1051 participants had a complete age BMI score. Ten of these participants were removed from the sample because they reported they were pregnant at the time. Additionally, one participant was added because they were only missing their midlife height; therefore, their age 18 height was used in the calculation. The final sample used in this analysis was 1042. Two hundred and eighty-six participants (268) completed both the self-report survey by age 37 and a comprehensive in-person health exam at ages 37 to 39 (March 24, 2017 to December 21, 2019), whereby their BMI was also computed. The in-person exam occurred at the Department of Preventive Medicine Research Clinic, Northwestern University Feinberg School of Medicine in Chicago, Illinois.

The correlation between the two BMI measures was high (r= .85), which supports the validity of the survey reports. Self-reported BMI was also highly correlated with the in-person body composition score (r = .65) and waist circumference (r = .78). The correlation between self-reported BMI and the in-person exam was similar for females and males (0.81 and 0.90) and for those in the bottom and top half of the self-report distribution (0.70 and 0.66).

**Covariates and Group Equivalence**

Based on administrative records from multiple sources and parent surveys,[1-4] 17 variables were included as model covariates. They were measured primarily from birth to age 3 as baseline characteristics. Two significant differences were detected between groups: CPC participants grew up in higher poverty neighborhoods and their parents had higher rates of high school completion (but not college attendance). Program and comparison groups were similar on a range of baseline characteristics at the age 37 follow up and at the beginning of the study. Selected baseline characteristics are provided at the end of this section. Breakdowns for women and men separately showed similar results. Rates of attrition and the characteristics of those missing at follow up were similar within and between groups, especially after accounting for the process of attrition (see section "Inverse Propensity Score Weighting").

A primary explanation for equivalence between groups on most indicators was that the comparison group included all children from five randomly selected schools (out of 27 citywide) in similar low-income neighborhoods that participated in the usual early childhood program at the time: full-day kindergarten without earlier preschool participation. All cohort participants enrolled in the Chicago Public School District beginning at ages 3 or 4 with nearly all comparison group participants entering in kindergarten. Fifteen percent of the comparison group attended Head Start preschool as part of the usual programming.[1,3] Although the focus of the current study is the preschool component, CPC services are provided up to 2nd or 3rd grade in 20 schools. Program expansion has occurred in Chicago and other Midwest districts.

Because the CPCs were opened in the highest poverty areas of the city, the neighborhoods surrounding the centers were generally more disadvantaged than other areas. This is shown in the select comparisons below. However, this provides a conservative bias in estimating impacts, since lower-resourced settings link to higher rates of educational and health problems as well as lower access to high-quality health services and preventive care. We controlled for this and other factors. In addition, these factors were also included in the estimated propensity score for addressing potential attrition bias (see below). At baseline, 50% of the CLS cohort (N = 520; 522 were below threshold) resided in high-poverty neighborhoods, defined as 40% or more of the neighborhood population (census tracks) at or below the federal poverty level. At the midlife follow up,

© 2021 Reynolds AJ et al. *JAMA Pediatrics*.

the percentage of the cohort currently residing in high-poverty neighborhoods was 26% (N = 275; 767 below threshold). Roughly two-thirds of the sample lived in Chicago at follow up.

CPC school-age participation (1st to 3rd grade) was included in the model (not a study focus) with 17 covariates. Those measured dichotomously were CPC school-age enrollment, Black, Female, eight sociodemographic family risk factors (e.g., parent dropped out of high school, family income 130% of the federal poverty line or below, single parent status, attended school in a low-income area), family risk indicator was imputed, received child welfare services, stressful home environment (retrospective report from participants), parent attended college, resided in high poverty neighborhood (40% or more of residents were at/below federal poverty level), and resided in neighborhood with relatively high human capital (10% or more of residents ages 25 and above had a 4-year college degree). Birth weight in pounds from the Illinois Department of Health was the only continuous measure.

A summary of group differences for select baselines characteristics is below. The original sample sizes for program and comparison groups were 989 and 550, respectively. P-values at .05 level are starred for both follow up and original samples (Descriptive statistics for the original sample are not shown). After adjustment for attrition, no differences in sample retention were found.

| Child or Family Characteristic | Prog. Group (n=689) | Comp. Group (n=353) | P-value | Original sample P-value |
|---|---|---|---|---|
| Percent of cohort in follow up (unadjusted) | 69.7 | 64.2 | .03* | -- |
| Percent of cohort in follow up (attrition adj) | 69.5 | 68.0 | .57 | -- |
| Percent women | 54.3 | 47.9 | .06 | .09 |
| Percent Black | 93.5 | 94.3 | .69 | .68 |
| Family risk index (0-7) by child's age 3 | 4.42 | 4.46 | .68 | .80 |
| Percent four or more family risk factors | 71.1 | 71.4 | .94 | .63 |
| Percent mother not completed high school | 49.8 | 56.9 | .03* | <.01* |
| Percent mother completed some college | 13.4 | 10.8 | .28 | .10 |
| Percent single parent by child's age 3 | 75.0 | 76.2 | .70 | .75 |
| Percent mother not employed | 65.6 | 64.0 | .63 | .29 |
| Percent ever reported receiving free lunch | 82.9 | 82.7 | .99 | .52 |
| Percent ever reported receiving AFDC | 60.4 | 61.2 | .84 | .24 |
| Percent having 4 or more children at home | 16.1 | 18.4 | .38 | .35 |
| Percentage of children in school area in low income families (<185% of poverty level) | 66.6 | 67.1 | .39 | .15 |
| Reside in neighborhood ≥40% pop. at poverty line | 56.2 | 37.1 | <.01* | <.01* |
| Percent mother was teen at child's birth | 14.7 | 16.1 | .52 | .39 |
| Birthweight in pounds | 6.83 | 6.72 | .21 | .26 |

**Inverse Propensity Score Weighting**

Following prior studies,[1,3] Inverse Propensity Score Weighting (IPW) was used to adjustment for potential attrition bias. Nearly 30% of the original cohort did not complete the midlife survey. IPW methods can reduced attrition bias arising from measurable factor influencing sample recovery status.[19] The regression model included the following weight variable:

$$W_i = 1/P_i$$

$$P_{i\,(SR)} = \text{Constant} + \boldsymbol{B_j}BD + \mathbf{B_j}HE + \mathbf{B}_jPR + \mathbf{B}_jSN + e$$

The predicted probabilities of sample recovery (SR; age 37 survey) were estimated by logistic regression (OLS regression also yields consistent estimates) with 31 input predictors hypothesized or known to be important. These included birth outcomes and demographics (BD), home environment (HE), program (PR), school, and neighborhood factors (SN). In the outcome regressions, this weight was applied such that individuals with higher weights were counted more heavily in program effect estimates (they have lower probabilities of responding to the adult survey). Those with lower weights were counted less. The weight

© 2021 Reynolds AJ et al. *JAMA Pediatrics*.

variable (W) ranged from 1.08 to 3.08 with mean of 1.5. Thus, cases at the mean were counted nearly 40% more than those in the lower range. Standard errors were adjusted for the weighted regressions. Analyses revealed that estimated program impacts were similar between IPW and non IPW models. The IPW procedure effectively adjusted for group differences in rates of sample recovery (see above select comparisons). Further information also is available.[6,7]

**Program Description**

The CPC program began in 1967 in four new centers on Chicago's westside (East and West Garfield Park, North Lawndale). These were, and remain today, the highest poverty neighborhoods in the city. This was the result of the landmark Elementary and Secondary Education Act of 1965 for which federal funding from Title I of the Act was used by the school district to open the centers. The Chicago Public School District was the first to use Title I for preschool and thereby established CPC as the second oldest (after Head Start) federally-funded preschool.[8]

The program was developed in response to three major problems facing Chicago schools: low rates of attendance, family disengagement with schools, and low student achievement.[8,9] The conceptual foundation is that well-being is a product of proximal and distal influences at multiple levels of contexts (individual, family, school, community) experienced during the entire early childhood period (ages 3 to 9). Although CPC began as a comprehensive preschool program, children received continuing services in kindergarten and the early grades the following year, resulting in the preschool to 3rd grade program that it is today. The program was modified as a school reform model in 2012 as part of expansion in and outside of Chicago funded by the U. S. Department of Education. Six core elements are implemented; effective learning experiences, collaborative leadership, aligned curriculum, parent involvement and engagement, professional development, and continuity and stability.[10] At present, there are 19 centers in Chicago.

CPC provides comprehensive, multi-systemic services in education and schooling, family support, health, and community outreach.[8-10] Under the direction of the Head Teacher at each site and in collaboration with the Principal, CPC enhances school readiness and achievement, promotes parent involvement and engagement in the school and community, and enhances socio-emotional learning with an emphasis on self-control, self-efficacy, and personal responsibility. Breakfasts and lunches are provided, school nurses work with families on site, and referrals to health centers, speech therapy and other supports are provided. CPCs are in a stand-alone school or center in which all children receive services. After a part-day program (3 hours, 5 days per week) at ages 3 and/or 4 in small classes with child-teacher ratios of 17:2, the K–3rd components provide reduced class sizes (maximum of 25), teacher aides for each class, health services, continued parent involvement opportunities, and enriched classroom environments for strengthening language and literacy, math, science, and social-emotional learning.

To promote wholistic well-being, including physical health, each center has a parent resource room and family program run by the Parent-Resource Teacher in collaboration with the School-Community Representative. The later conducts home visits, engages parents in the school, mobilizes resources in the community and provides referrals to health, employment and job training, and related services. Parent workshops and trainings are a predominant element of the program, and they most frequently include child development, health literacy, nutrition, financial literacy, and personal development topics. GED courses are often provided on site and parents volunteer in the classroom and in community organizations. Given the physically located resource room in the centers, peer support among parents and family members is another key feature.

Based on the goals and foci of the program, participants' experiences, and impacts to date,[8-13] the program is expected to promote healthy body mass and reduce obesity over the life course through enhancing for mechanisms of change:

a. educational success and attainment
b. self-control and self-efficacy behaviors
c. health literacy and practices
d. social support and engagement.

© 2021 Reynolds AJ et al. *JAMA Pediatrics*.

**eReferences**

1. Reynolds, AJ., Ou, S, Temple, JA. (2018). A multicomponent, preschool to 3rd grade preventive intervention and educational attainment at 35 years of age. *JAMA Pediatrics.* 2018;172*(3),* 247-256.

2. Reynolds AJ, Temple JA, Robertson, DL, Mann, EA. (2001). Long-term effects of an early childhood intervention on educational achievement and juvenile arrest: A 15-year follow-up of low-income children in public schools. *JAMA, 285*(18), 2339-2346.

3. Reynolds, AJ, Temple, JA, Ou, S, Arteaga, IA, White BA. (2011). School-based early childhood education and age-28 well-being: Effects by timing, dosage, and subgroups. *Science 333*(6040), 360-364.

4. Chicago Longitudinal Study. *CLS user's guide: A study of children in the Chicago Public Schools*. Minneapolis: University of Minnesota, Institute of Child Development, 2005.

5. Imbens GW, Wooldridge JM. Recent developments in the econometrics of program evaluation. *Journal of Economic Literature.* 2009*; 47*(1), 5-86.

6. Chicago Longitudinal Study: Background and supporting information. Minneapolis: Human Capital Research Collaborative, University of Minnesota, 2020. http://innovation.umn.edu/cls/wp-content/uploads/sites/23/2020/10/cls-background-bmi-102020.pdf

7. Hales CM, Carroll MD, Fryer CD, Ogden CL. Prevalence of obesity and severe obesity among adults: United States, 2017-2018. *NCHS Data Brief No. 360*. National Center for Health Statistics, 2020. https://www.cdc.gov/nchs/products/databriefs/db360.htm

8. Reynolds AJ. *Success in early intervention: The Chicago Child-Parent Center*. Lincoln: University of Nebraska Press, 2000.

9. Sullivan, L. M. *Let us not underestimate the children*. Glenview, IL: Scott Foresman, 1971.

10. Reynolds AJ, Hayakawa M, Candee, AJ, Englund, MM. *CPC P-3 program manual: Child-Parent Center Preschool-3rd Grade Program.* Minneapolis, MN: Human Capital Research Collaborative, University of Minnesota, 2016.

11. Reynolds AJ, Temple JA, White, BA, Ou S, Robertson DL. (2011). Age-26 cost-benefit analysis of the Child-Parent Center early education program. *Child Development*, *82*(1), 379-404.

*12.* Reynolds AJ, Ou, S. (2011). Paths of effects from preschool to adult well-being: A confirmatory analysis of the Child-Parent Center Program. *Child Development,* 82(2), 555-582.

13. Reynolds AJ, Temple JA, Ou S, et al (2007) Effects of a school-based, early childhood intervention on adult health and well-being: A 19-year follow-up of low-income families. *Arch Pediat Adol Med.* 161(8):730-739.

© 2021 Reynolds AJ et al. *JAMA Pediatrics*.
